# Intraovarian PRP injection improves oocyte quality and early embryo development in mouse models of chemotherapy-induced diminished ovarian reserve

**DOI:** 10.18632/aging.206099

**Published:** 2024-09-13

**Authors:** Mauro Cozzolino, Yagmur Ergun, Denis A. Seli, Sonia Herraiz

**Affiliations:** 1 IVIRMA Global Research Alliance, IVI Foundation-IIS la Fe, Valencia 46026, Spain; 2 Department of Obstetrics, Gynecology and Reproductive Sciences, New Heaven, CT 06510, USA; 3 IVIRMA Global Research Alliance, IVIRMA Roma, Rome 00197, Italy

**Keywords:** platelet-rich plasma ovarian injection, poor ovarian reserve, premature ovarian insufficiency

## Abstract

Intraovarian injection of platelet-rich plasma (PRP) has been recently proposed, with encouraging results to provide an alternative option to patients diagnosed with POR or POI. However, the broad spectrum of PRP effects on the reproductive function and the mechanisms of action in follicular activation, response to stimulation, and embryo quality have not yet been studied. In this study, we first induced poor ovarian reserve (POR) and premature ovarian insufficiency (POI) ovarian phenotypes in CD1 mice undergoing PRP or sham intraovarian injection.

PRP administration reduced those alterations induced by chemotherapy in ovarian stroma and follicle morphology in both the POR and POI conditions. After ovarian stimulation, we found that PRP did not modify the MII-oocyte yield. Nevertheless, the amount of obtained 2-cell embryos and fertilization rate were increased, being especially relevant for the POI model. Further *in vitro* embryo culture led to improved blastocyst formation rates and higher numbers of good quality blastocysts in PRP vs. sham females in both the POR and POI conditions. These positive results of PRP injection were also validated in the C57Bl/6 stain.

Altogether, our findings suggest a possible effect on oocyte and embryo quality. This effect is likely due to the increase of local paracrine signaling through the released growth factors in PRP-treated ovaries.

## INTRODUCTION

Ovarian aging is associated with a progressive physiological decline in the quantity and quality of the oocytes [1]. In the past two decades, demographic changes associated with a delay in the age of childbearing contributed to the increase in the number of individuals needing fertility treatments due to diminished number of oocytes available in the ovary [2].

In the clinical setting, diminished reserve of oocytes in a patient is described using two distinct diagnostic terms, representing different levels of severity. One of them, poor ovarian response (POR, also called diminished ovarian reserve (DOR)), is a milder but clinically relevant entity affecting 15% of women undergoing IVF treatment in the United States [3]. According to the ESHRE BOLOGNA consensus, POR diagnosis requires 2 of the following criteria: ≥40 years of age, prior poor ovarian response to ovarian stimulation, and abnormal ovarian reserve test [4]. Patients diagnosed as POR using the Bologna criteria generate a lower number of embryos per cycle and have lower cumulative pregnancy rates using conventional assisted reproductive technologies (ART) [5]. Conversely, primary ovarian insufficiency (POI), affects 1% of reproductive-age women and according to ESHRE criteria represents a more severe decrease in ovarian reserve in younger women (<40 years old), with oligomenorrhea/amenorrhea for at least 4 months, and serum follicle-stimulating hormone (FSH) levels ≥25 IU/ml [6]. At present time, the only practical treatment for women with POI is oocyte donation.

To provide an alternative treatment option to patients diagnosed with POR or POI, intraovarian injection of platelet-rich plasma (PRP) has been recently proposed, with encouraging results [7–11]. PRP consists of a high concentration of platelets found in plasma obtained after centrifugation of peripheral blood [12] and carries more than 800 types of proteins, molecules, cytokines, hormones, and chemoattractants [13]. The activation of platelets induces the release of a variety of biologically active proteins, which stimulate cell proliferation, growth, and differentiation.

While these effects may underlie the encouraging outcomes observed in patients treated with autologous intraovarian PRP injection, the mechanism of action of PRP in follicular activation has not yet been studied. Thus, the current study aimed to characterize the protein content of PRP and investigate whether PRP improves reproductive function in different mouse strains with chemotherapy-induced POR and POI.

## MATERIALS AND METHODS

### Animal breeding and genotyping

All animal care, breeding and experiments were conducted according to the Yale University Animal Research Requirements, and the protocols were approved by Institutional Animal Care and Use Committee (Protocol # 2020-11207).

A total of 65 eight-week-old CD1 female mice were purchased from Charles River Laboratories and 35 eight-week-old C57BL6 female mice from Yale animal facility.

During the entire experiment, all the mice were fed a standard diet ad libitum and housed in a 12:12-hour light-dark cycle.

### Study design

**Figure 1:**
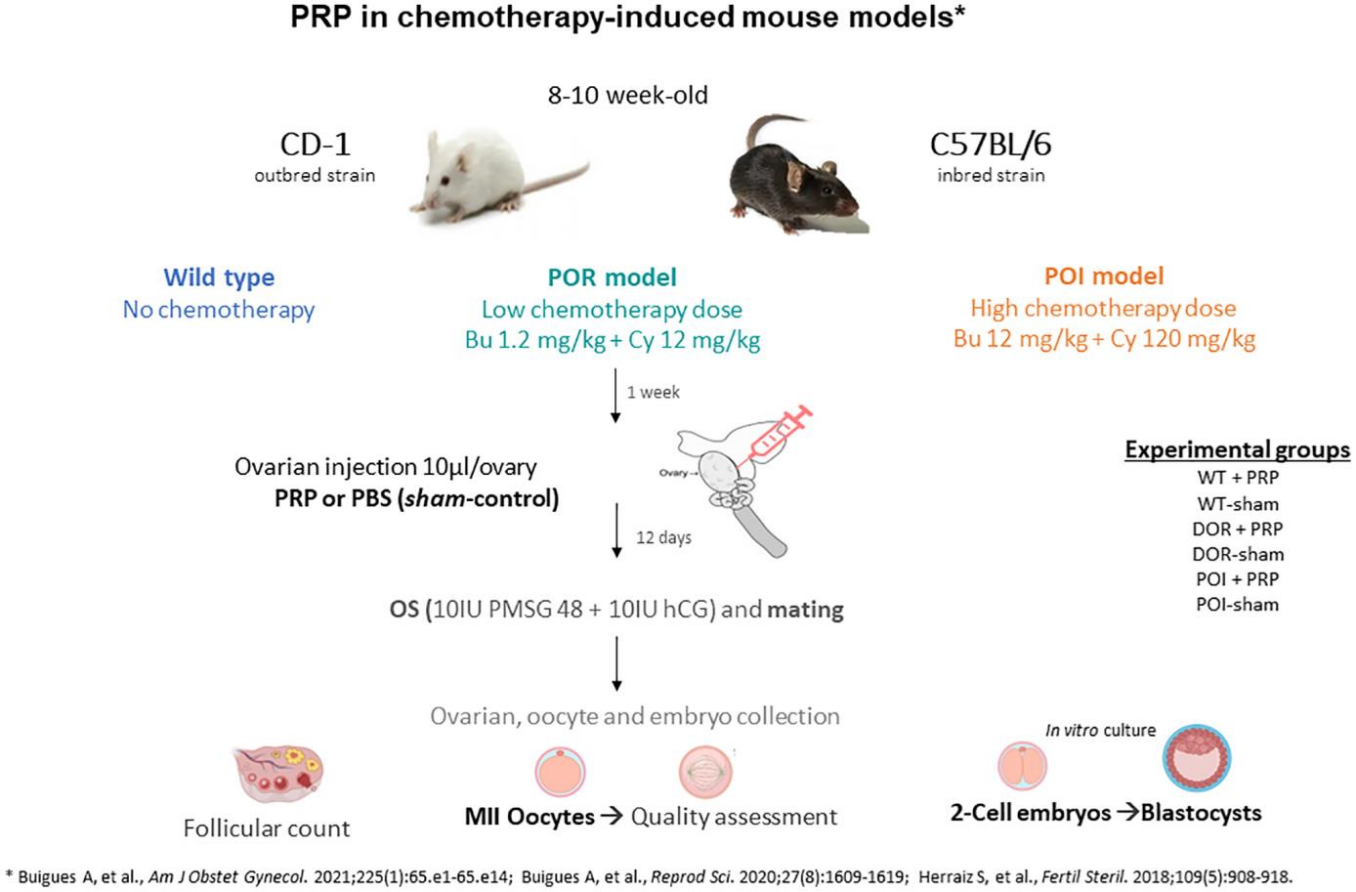
**Experimental design scheme for testing the regenerative effects of platelet rich plasma (PRP) in chemotherapy-induced ovarian damage mouse models using CD1 and C57BL/6 strains.** Abbreviations: POR: poor ovarian reserve; POI: premature ovarian insufficiency; OS: ovarian stimulation; PBS: phosphate buffered saline.

In this study, we used well established mouse models of POR and POI [14] in CD1 and C57/BL6 female mice (8-week-old) by intraperitoneal injection of two different doses of cyclophosphamide and busulphan (POR: 12 mg/kg + 1.2 mg/kg and POI: 120 mg/kg + 12 mg/kg, respectively) while non treated females were used as controls (*n* = 20 for each treatment group) [15–17]. One week after chemotherapy, once the ovarian damage was established, animals from POR, POI and control groups were randomized to receive an intraovarian injection of 10 µl of PBS (sham injection) or PRP in both ovaries (Figure 1). A total of 60 CD1 mice (*n* = 10 in each group) were randomized to the following experimental conditions: (1) Wild-type (WT)-sham, (2) WT-PRP, (3) POR-sham, (4) POR-PRP, (5) POI-sham, and (6) POI-PRP.

Fourteen days after receiving PRP or sham intervention, mice underwent controlled ovarian hyperstimulation (COS) with 10 IU of pregnant mare serum gonadotropin (PMSG, Sigma-Aldrich, USA) followed, 48 h later, by 10 IU of human chorionic gonadotropin (hCG, Sigma-Aldrich, USA) and were mated with wild-type males. Females were euthanized 36 h after hCG injection by cervical dislocation to collect recovered ovulated oocytes and 2-cell embryos. Immediately after collection, oocytes and embryos were expelled from the oviduct using a 30 G needle loaded with global collect medium (GCOL-100). Day 2 embryos were then *in vitro* cultured until the blastocyst stage with Sage 1-step medium (both from CooperSurgical Fertility and Genomic Solutions, Ballerup, Denmark) at 37°C with 5% O_2_, 6% CO_2_ and 89% N_2_. Ovaries were also collected and immediately fixed in neutral-buffered formalin to analyze ovarian reserve and follicular development (*n* = 4/group). The collection of 2-cell embryos and oocytes from oviducts was first performed and then the ovaries were fixed in formalin for histomorphometric analyses.

Experiments were then repeated in 8-week-old C57/BL6 females (*n* = 5 in each group; a total of 30) to validate the reproductive outcomes after COS in control, POR and POI groups after receiving PRP or sham injection.

Finally, PRP from both the CD1 and C57/BL6 strains was characterized by LC-MS/MS technique to characterize proteomic composition of PRP and determine if it was different among strains.

### PRP preparation

Total blood was collected from additional CD1 and C57/BL6 females (*n* = 5/each) by aortic puncture after sacrifice (~1 mL/female). Collected samples were centrifuged at 1500 rpm for 8 minutes to separate plasma from cell detritus. Then, total plasma was centrifuged for 15 min at 2000 rpm to isolate the upper 1/3 fraction of the plasma volume, corresponding to the platelet-rich plasma fraction (PRP, 150–175 µl per female), from the platelet-poor fraction. Finally, the freeze-thaw technique was used to promote PRP activation through maximal release of α-granule growth factors as previously described [18–20].

### Intraovarian injection procedure

Briefly, mice were anesthetized using isoflurane, before premedication with buprenorphine after incision of the back skin and opening of the peritoneum to identify and expose the ovaries bilaterally. Then, 10 µl of PRP or PBS was injected using an insulin syringe of 0.5 ml BD micro-fine plus 31 G (BD Diagnostics, Madrid, Spain) into the center of each ovary. Layered closure of the abdominal wall was performed. The animals were kept under observation for 1 h after the administration of antalgic therapy.

### Assessment of ovarian reserve and folliculogenesis

To obtain hematoxylin and eosin-stained (H&E) sections for follicular counts, ovaries were collected and fixed in 4% paraformaldehyde (Sigma-Aldrich, St. Louis, MO, USA) at 4°C overnight, and kept in 70% ethanol until it was processed. Dehydration, embedding, and sectioning (5 µm) steps were performed, and sections were stained with H&E. Every 5th section was assessed for follicles containing oocytes with a visible nucleus. To avoid double-counting, normal follicles were only counted when the oocyte nucleus was present in the section. Sections from both ovaries in the mice were counted to establish the total number of follicles. Follicles were categorized as primordial, primary, secondary, early antral, and antral follicles. Primordial follicles were described as surrounded by a single layer of flat and squamous granulosa cells. Primary follicles were defined as a single layer of cuboidal granulosa cells surrounding the oocyte whereas secondary follicles were considered as oocytes surrounded by two or three layers of cuboidal granulosa cells with no visible antrum. Early antral follicles show the formation of antrum cavity and are usually surrounded by four or more layers of granulosa cells. Antral follicles were described as follicles containing a clearly defined single antral space. Follicles were considered morphologically abnormal when degeneration and necrosis of granulosa cells and/or oocytes were observed. Morphologically normal vs. atretic follicles were defined on the basis of the following criteria: basement membrane integrity, cellular density, presence or absence of pyknotic granulosa cell nuclei, oocyte integrity [21, 22]. All H&E sections were examined by 2 observers (M.C. and S.H.).

### Collection and analysis of MII oocytes, 2-cell embryos and blastocysts

Oviducts were harvested and cut between the ovary and the uterus. Oviducts were then placed in 1 ml medium (Global Collect, GCOL-100, CooperSurgical) in a small Petri dish. The embryos and oocytes from the oviduct were expelled by inserting a 30-gauge needle into the infundibulum and flushing 60 µl of medium through the oviduct. Oocytes and embryos were collected with a stripper and a 170 µm capillary and transferred to a 35 mm plate with drops of Flushing Medium (Flushing Medium, 10845060A, CooperSurgical) for their maintenance and classification, using a binocular loupe. A morphologically normal or viable MII oocyte was defined as one with a homogeneous ooplasm, a proportionate, well-defined, and regular zona pellucida and a non-enlarged perivitelline space with a single polar corpuscle. A morphologically normal 2-cell embryo was defined as one with two symmetrical, non-fragmented cells and a proportionate zona pellucida.

Isolated 2-cell embryos were then cultured in 100 µl of medium SAGE 1-Step^™^ (SAGE 1-Step^™^, with HSA and phenol red, 67010060A, CooperSurgical) at 37° degree at 5% CO_2_ during 72 h to the blastocyst stage. At that time, embryos were classified according to morphological criteria using a binocular loupe. Fully developed blastocysts, presenting morphologically normal inner cell mass and trophectoderm were defined as high-quality blastocysts.

### PRP proteome characterization

#### In solution protein digestion

The platelet-rich plasma samples were centrifuged at 14,600 g at 4ºC for 10 minutes to pull any debris down. Aliquots of 10 µL (~100 µg) of the supernatant were taken and 90 µL of water was added. The proteins were precipitated utilizing an acetone precipitation procedure. The resulting pellets were dissolved and denatured in 50 µl 8 M urea, 0.4 M ammonium bicarbonate. The proteins were reduced by adding 5 µl 45 mM dithiothreitol (Pierce Thermo Scientific #20290, USA), incubated at 37ºC for 20 minutes, and then alkylated with the addition of 5 µl 100 mM iodoacetamide (Sigma-Aldrich #I1149, USA) with incubation in the dark at room temperature for 30 minutes. The urea concentration was adjusted to 2 M by the addition of water. Samples were then enzymatically digested with 4 µg LysC at 37ºC for 16 hours followed by trypsin digestion (4 µg, Promega Seq. Grade Mod. Trypsin, #V5113) at 37ºC for 7 hours. Samples were desalted using BioPureSPN PROTO 300 C18 macro spin columns (The Nest Group, #HMM S18V, USA) following the manufacturer’s directions with peptides eluted with 0.1% TFA, 80% acetonitrile. The eluted sample was speedvaced dry and stored at −80ºC until data collection. Peptides were dissolved in MS loading buffer (2% acetonitrile, 0.2% trifluoroacetic acid). A nanodrop measurement (Thermo Scientific Nanodrop 2000 UV-Vis Spectrophotometer) determined protein concentrations (A260/A280). Each sample was then further diluted with MS loading buffer to 0.06 µg/µl, with 0.3 ug (5 µl) injected for LC-MS/MS analysis.

#### LC-MS/MS on the Thermo Scientific orbitrap fusion

LC-MS/MS analysis was performed on a Thermo Scientific Orbitrap Fusion equipped with a Waters nanoAcquity UPLC system utilizing a binary solvent system (Buffer A: 100% water, 0.1% formic acid; Buffer B: 100% acetonitrile, 0.1% formic acid). Trapping was performed at 5 µl/min, 99.5% Buffer A for 3 min using a Waters Symmetry^®^ C18 180 µm × 20 mm trap column. Peptides were separated using an ACQUITY UPLC PST (BEH) C18 nanoACQUITY Column 1.7 µm, 75 µm × 250 mm (37ºC) and eluted at 300 nl/min with the following gradient: 3% buffer B at initial conditions; 5% B at 5 minutes; 20% B at 125 minutes; 35% B at 170 minutes; 97% B at 175 minutes; 97% B at 180 min; return to initial conditions at 182 minutes. MS was acquired in the Orbitrap in profile mode over the 350–1,550 m/z range using wide quadrapole isolation, 1 microscan, 120,000 resolutions, AGC target of 4E5, and a maximum injection time of 60 ms. Data-dependent MS/MS were collected in top speed mode with a 3 s cycle time on species with an intensity threshold of 5E4, charge states 2–8, and peptide monoisotopic precursor selection preferred. Dynamic exclusion was set to 30 seconds. Data-dependent MS/MS were acquired in the Orbitrap in centroid mode using quadrupole isolation with a 1.6Da isolation window, HCD activation with a collision energy of 28%, 1 microscan, 60,000 resolution, AGC target of 1E5, maximum injection time of 110 ms.

#### Peptide identification

Data were analyzed using Proteome Discoverer (version 2.5) (Thermo Scientific, USA) software and searched in-house using the Mascot algorithm (version 2.8.0) (Matrix Science). The data were searched against the Swissprotein database with taxonomy restricted to *Mus musculus* (17,097 sequences). Search parameters used were trypsin digestion with up to 2 missed cleavages; peptide mass tolerance of 10 ppm; MS/MS fragment tolerance of +0.02 Da; Fixed modification of carbamidomethylated cysteine and variable modifications of methionine oxidation, acetylated lysine and phosphorylation on serine, threonine and tyrosine. Normal and decoy database searches were searched, with the confidence level set to 95% (*p* < 0.05). Scaffold v5.1.2 (Proteome Software Inc., Portland, OR, USA) was used to validate MS/MS based peptide and protein identifications. Peptide identifications were accepted if they could be established at greater than 95.0% probability. Protein identifications were accepted if they could be established at greater than 99.0% probability (assigned by the Protein Prophet algorithm) and contained at least 2 identified peptides. Proteins that contained similar peptides and could not be differentiated based on MS/MS analysis alone were grouped to satisfy the principles of parsimony. Proteins sharing significant peptide evidence were grouped into clusters.

### Statistics

Kruskal-Wallis tests followed by Mann-Whitney *U*-tests for two-by-two comparisons, were performed in GraphPad Prism v.8.12 (GraphPad Software, San Diego, CA, USA). *P*-values < 0.05 were considered statistically significant.

## RESULTS

### Chemotherapy-induced POR and POI mouse models: ovarian reserve and reproductive outcomes

At the time of ovarian stimulation and sacrifice, CD1 females with chemotherapy-induced ovarian damage did not show any major clinical signs associated with ovarian surgery and PRP/saline injection. No differences were detected when body mass was compared between PRP-treated and control mice for any of the ovarian conditions tested. Nevertheless, chemotherapy administration induced changes in the ovarian stroma such as increased fibrosis, enlarged blood vessels and different grades of tissue degeneration according to dosage (Supplementary Figure 1). These changes were accompanied by presence of apoptotic granulosa cells and increased follicular atresia, especially from secondary and late preantral growing follicles in CD1 ovarian samples. PRP administration reduced these deleterious effects of chemotherapy in stroma and follicles in the POR and POI animals.

The total follicle number decreased in a dose-dependent manner in POR and POI females in response to chemotherapy, regardless of PRP administration (Supplementary Figure 2A, 2B). The CD1 strain, which is known for their fertility and high number of oocyte generation showed a decline in primordial and total number of follicles, however, the difference was not statistically significant due to high variability. Additional validation was successfully done in the C57/BL6 strain, where significant reduction in follicle numbers were demonstrated in both POR and POI groups (Supplementary Table 1).

When follicular counts were compared between PRP and sham treated animals for each ovarian condition, PRP administration induced an increase in all follicular populations when compared to own control in both wild type and POI models. Interestingly, this trend was not observed in the POR model (see Table 1 and Supplementary Figure 2B). To exclude possible deleterious effects of PRP injection in healthy ovaries, follicular counts were also performed in the wild type females, with no statistically significant differences induced by PRP injection in young healthy ovaries compared to sham-saline. The absence of effects when the number of primordial, primary, secondary, early antral, and antral follicles in healthy ovaries was reassuring for the safety of the technique.

**Table 1:** Follicle development was assessed in the ovaries of 2-month-old mice after hyperstimulation.

**CD1 STRAIN**	**WT-C**	**WT-PRP**	**POR-C**	**POR-PRP**	**POI-C**	**POI-PRP**
**Primordial**	52.5 ± 34.2	59.3 ± 34.1	50.6 ± 23.7	53.0 ± 44.5	18.0±20.5	25.5 ± 20.6
**Primary**	101.6 ± 17.6	122.8 ± 15.9	111.6 ± 27.5	70.3 ± 25.9	45.6 ± 45.1	79.9 ± 21.7
**Secondary**	111.3 ± 37.0	111.1 ± 20.0	98.8 ± 17.9	74.3 ± 21.5	53.8 ± 38.8	69.8 ± 27.9
**Early antral**	64.6 ± 19.6	86.6 ± 42.6	54.5 ± 21.5	36.4 ± 8.9	40.1 ± 32	53.4 ± 14.6
**Antral**	15.9 ± 7.7	16.9 ± 10.8	17.0 ± 4.4	13.3 ± 1.7	16.5 ± 2.7	17.8 ± 6.7
**Total**	345.9 ± 76.6	396.6 ± 66.2	332.5 ± 23.5	247.1 ± 90.0	174.0 ± 136.3	246.3 ± 80.9

Data were obtained from 5 mice per group and are presented as Mean ± SD.

**Figure 2:**
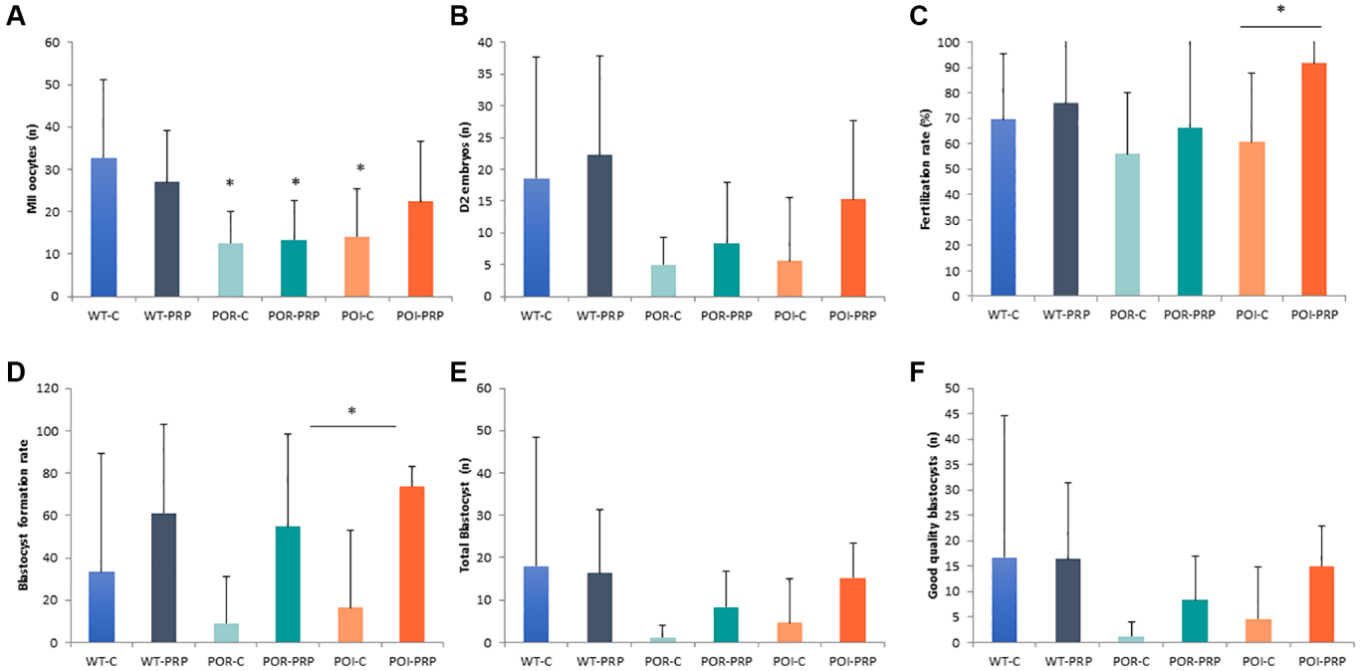
**Reproductive outcomes in chemotherapy-induced POR and POI mice model using the CD1 strain.** (**A**) MII oocytes (*n*) and (**B**) Day 2 embryos collected from the oviduct. (**C**) Fertilization rate. (**D**) Blastocyst formation rate (%), (**E**) total amount of blastocyst obtained after *in vitro* embryo culture. (**F**) Good quality blastocysts. Data are presented as mean ± Standard Deviation (SD). ^*^p < 0.05, using Kruskal-Wallis in the comparison as indicated and Mann-Whitney *U*-tests for two-by-two comparisons.

After ovarian stimulation, we found that both the POR and POI models showed reduced MII-oocyte numbers when compared to the wild-type controls (*p* < 0.05) due to chemotherapy administration, confirming the POR and POI ovarian phenotypes of our models (Figure 2A).

PRP injection did not modify the number of recovered MII oocytes in wild-type females (27 ± 12.2 vs. 32.7 ± 18.4), or POR females (13.3 ± 9.3 vs. 12.6 ± 7.6), compared to their own sham-controls (Figure 2A). However, in the POI mice, PRP increased the number of collected mature oocytes (22.4 ± 14.2 vs. 14.1 ± 11.3). These results were mirrored by the amount of obtained 2-cell embryos and fertilization rates at collection, being especially relevant for the POI condition (15.3 ± 15.3 vs. 5.6 ± 10.1 Day 2 embryos, *p* < 0.05 and 91.6 ± 16.6% vs. 60.6 ± 27.2%, *p* = 0.019, respectively), where an increased percentage of properly fertilized 2-cell embryos were collected from the oviducts (*p* = 0.026) (Figure 2B, 2C and Supplementary Figure 3).

After *in vitro* embryo culture, PRP slightly increased the number of blastocysts in wild type and POR animals, with a significant 3-fold increase in the POI condition when compared to POI control females (15.2 ± 8.3 vs. 4.6 ± 10.2 blastocysts, *p* < 0.05) (Figure 2D, 2E).

These improvements were accompanied by a positive effect of PRP in oocyte and embryo quality in the three conditions, where improved blastocyst formation rates (61.3 ± 41.2 vs. 33.3 ± 56.2; 54.7 ± 43.9 vs. 9.0 ± 22.0; 73.8 ± 9.3 vs. 16.4 ± 36.7; *p* = NS, *p* = NS and *p* = 0.045, respectively) led to higher numbers of good quality *in vitro* cultured blastocysts when compared to non-treated females in the POR and POI conditions (8.3 ± 8.6 vs. 1.2 ± 2.9, *p* = NS; and 15.0 ± 7.9 vs. 4.6 ± 10.3, *p* = 0.03, respectively) (Figure 2D–2F and Supplementary Figure 2). Overall, PRP injection reduced the number of bad-quality embryos during *in vitro* culture when compared to non-treated females in the three conditions (wild type: 32.7 ± 44.3% vs. 68.7 ± 51.5%, *p* = NS; POR: 43.2 ± 44.5% vs. 90.3 ± 23.8%, *p* = NS; 15.8 ± 9.7% vs. 82.3 ± 39.6%, *p* = 0.04).

### Result validation in the C57Bl/6 strain: response to COS and fertility outcomes

**Figure 3:**
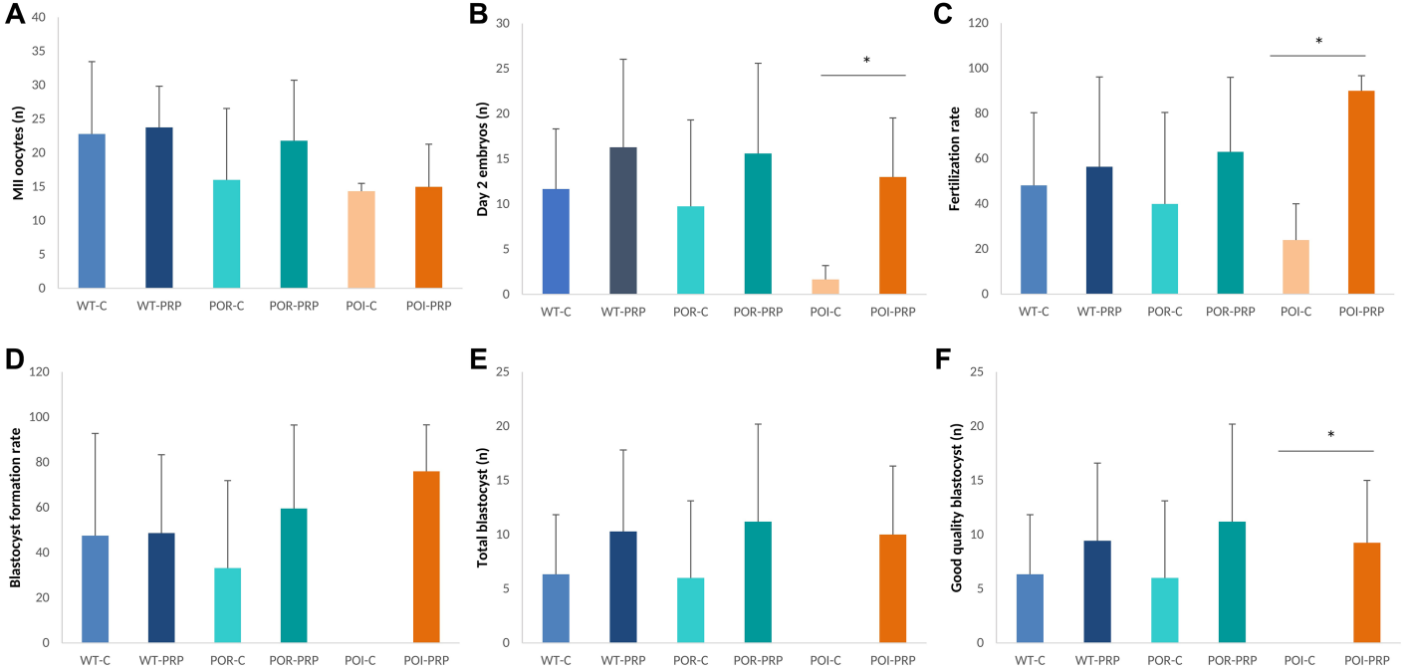
**Reproductive outcomes in chemotherapy-induced POR and POI mice models using the C57BL/6.** (**A**) MII oocytes (*n*) and (**B**) Day 2 embryos collected from the oviduct. (**C**) Fertilization rate. (**D**) Blastocyst formation rate (%), (**E**) total amount of blastocyst obtained after *in vitro* embryo culture. (**F**) Good quality blastocysts. Data are presented as mean ± SD. ^*^p < 0.05, using Kruskal-Wallis in the comparison as indicated and Mann-Whitney *U*-tests for two-by-two comparisons.

In order to validate our experimental models and the effects of PRP intraovarian injection, all the reproductive outcomes observed in the CD1 model were also assessed by using the C57BL/6 strain. In this case, we also observed that PRP injection did not modify the quantitative response to COS, measured as MII-oocyte yield, with no differences detected between PRP and sham controls in any of the three conditions (Figure 3A). However, positive effects of PRP injection were observed in all those parameters related to oocyte and embryo quality, and early embryo development, especially in the POI model (Figure 3B, 3C). Indeed, PRP-POI treated animals showed decreased percentage of bad quality MII oocytes at collection (1.8 ± 3.6% vs. 4.4 ± 7.7%; *p* = 0.008), increased numbers of 2-cell embryos (13.3 ± 5.1 vs. 3.3 ± 2.1, *p* = 0.049) and improved fertilization rate (90.1 ± 6.7 vs. 23.9 ± 16.1, *p* = 0.008) (Figure 3B, 3C); while reduced the percentage of bad quality embryos (28.3 ± 23.2% vs. 66.7 ± 57.7%, <0.05; Supplementary Figure 4) at the collection when compared to sham-POI females.

These outcomes led to increased blastocyst formation rates (46.0 ± 20.6% vs. 0.0 ± 0.0%), numbers of total blastocysts (10.0 ± 6.3 vs. 0.0 ± 0.0), and good quality blastocysts (9.3 ± 5.7 vs. 0.0 ± 0.0) after *in vitro* embryo culture in the POI-PRP mice compared to POI-sham controls (Figure 3D–3F), where no blastocysts were obtained in any case. Interestingly, several of these benefits were also observed in POR-PRP treated mice, although statistically significant differences were not reached due to the high variability observed within experimental groups.

### Proteomic composition of PRP from CD1 and C57BL/6 mouse strains

Based on the absence/presence profile, 241 proteins were identified in both C57BL/6 and CD1 mice, while an additional 56 proteins were identified only in C57BL/6 and 26 proteins only in the CD1 strain. Quantitative analysis revealed that 21 proteins were higher in CD1 mice, and 33 proteins were higher in C57BL/6 mice. For most proteins found in both strains, expression levels were not different between CD1 and C57BL/6 mice, including hepatocyte growth factor (HGF), vascular endothelial growth factor (VEGF), epidermal growth factor (EGF), and transforming growth factor-beta-induced protein.

**Figure 4:**
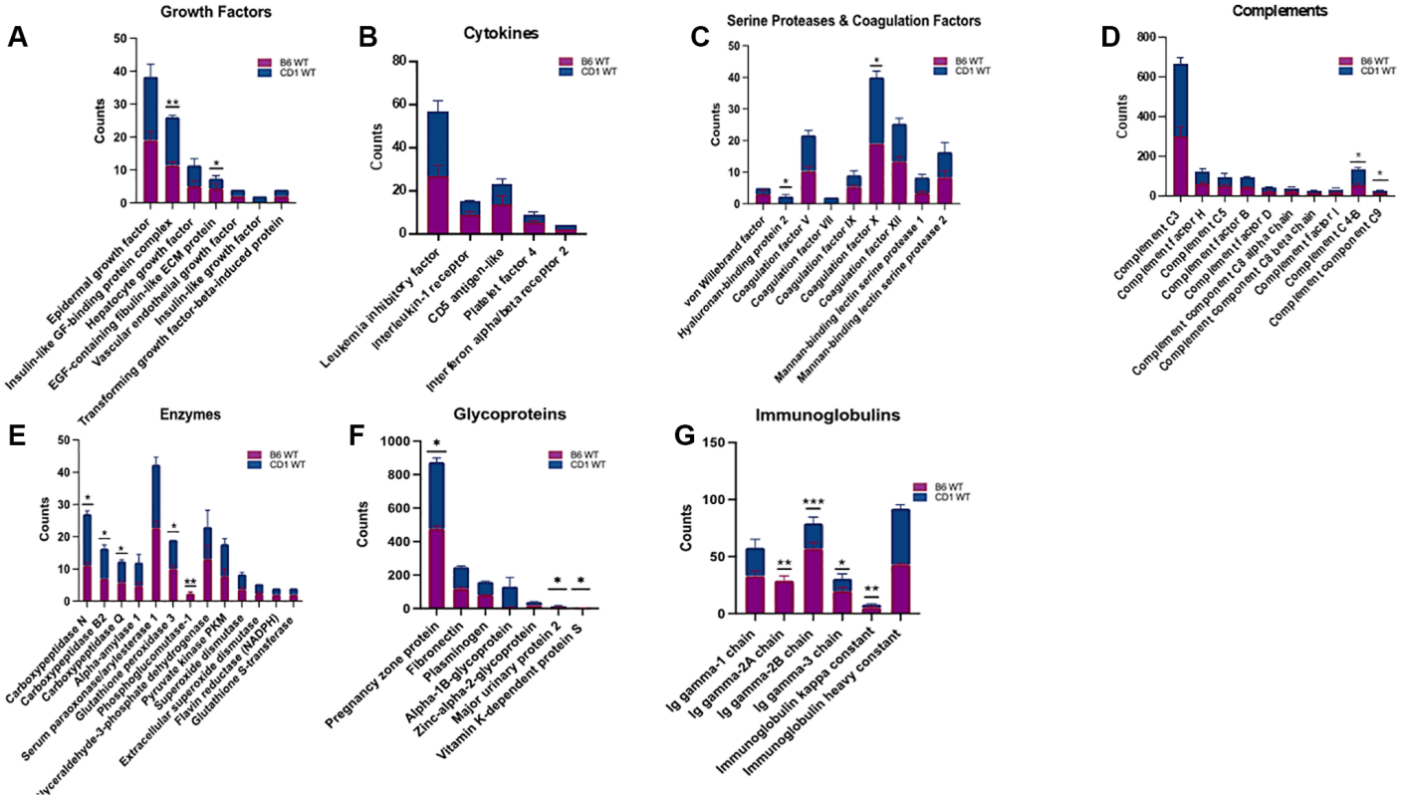
**Assessment and grouping of protein content of platelet rich plasma (PRP) among two strains of mice.** Different protein contents were found within B6 and CD1 wild-type mice strains. The proteins in PRP were categorized as Growth Factors (**A**), Cytokines (**B**), Serine Proteases and Coagulation Factors (**C**), Complements (**D**), Enzymes (**E**), Glycoproteins (**F**), and Immunoglobulins (**G**). The blue part of the bars represents CD1, and the purple bar represents the B6 strains. *B6: C57BL/6*. ^*^p < 0.05, ^**^p < 0.01, ^***^p < 0.001

When growth factors were analyzed, PRP from C57BL/6 mice had a higher amount of EGF-containing fibulin like extracellular matrix protein (4.3 ± 0.8 vs. 3 ± 0.5, *p* < 0.05), and insulin-like GF-binding protein complex (14.6 ± 0.33 vs. 11.3 ± 0.66) (Figure 4A – Growth Factor Graph). Leukemia inhibitory factor (LIF), interleukin 1 receptor (IL1R), Platelet factor 4, CD5 antigen-like protein, and interferon alpha/beta receptor 2 from cytokines showed insignificant differences (Figure 4B – Cytokines Graph).

PRP from CD1 mice contained insignificant differences of von Willebrand factor, Coagulation factor V, VII, IX, XII, Mannan binding lectin serine protease 1 and 2, and a significantly a higher amount of Hyaluronan-binding protein 2 (2.33 ± 0.33 vs. 0 ± 0 *p* < 0.05) and coagulation factor × (21.0 ± 1.15 vs. 19 ± 0, *p* < 0.05) (Figure 4C – Serine Proteases and Coagulation Graph).

Expression levels of the following complements were not different between CD1 and C57BL/6 mice: complement factor H, complement C5, complement factor B, complement factor D, complement component C8 alpha chain, beta chain and complement factor I. Whereas, complement C4-B (77.66 ± 4.91 vs. 55.33 ± 4.91 *p* < 0.05) and complement component C9 (15 ± 1.15 vs. 12.33 ± 1.20 *p* < 0.05) were significantly higher in CD1 mice (Figure 4D – Complements Graph).

Among enzymes, alpha-amylase 1, serum paraoxonase/arylesterase 1, glyceraldehyde-3-phosphate dehydrogenase, pyruvate kinase PKM, superoxide dismutase, extracellular superoxide dismutase, flavin reductase (NADPH), glutathione S-transferase showed insignificant difference between C57BL/6 and CD1 mice. Carboxypeptidase N, carboxypeptidase B2, and carboxypeptidase Q, glutathione peroxidase 3, and phosphoglucomutase-1 (16 ± 0.5 vs. 11 ± 0.5 *p* < 0.05, 9.33 ± 0.6 vs. 7 ± 0 *p* < 0.05, 6.66 ± 0.33 vs. 5.66 ± 0.33 *p* < 0.05, 9 ± 0.0 vs. 10 ± 0.57 *p* < 0.05, and 0 ± 0 vs. 2.3 ± 0.33 *p* < 0.01 respectively) were significantly different in CD1 mice compared to C57BL/6 strain (Figure 4E – Enzymes Graph).

Expression levels of the following glycoproteins were not different between CD1 and C57BL/6 mice: fibronectin, plasminogen, alpha-1B-Glycoprotein, zinc-alpha-2- glycoprotein whereas pregnancy zone protein (477 ± 0.88 vs. 398 ± 9.71, *p* < 0.05), major urinary protein 2 (9.33 ± 1.20 vs. 7 ± 1, *p* < 0.05), and vitamin K-dependent protein S (2 ± 0 vs. 3.33 ± 3.33, *p* < 0.05) were significantly different between PRP of CD1 and C57BL/6 strains (Figure 4F – Glycoproteins Graph).

Among immunoglobulins, Ig gamma-1 chain and Ig heavy constant were found to be similar between the two strains, when Ig gamma-2A chain (24.66 ± 4.33 vs. 33 ± 2.51, *p* < 0.01), Ig gamma-2B chain (22 ± 3.21 vs. 57 ± 3.21, *p* < 0.001), Ig gamma-3 chain (7.33 ± 4.05 vs. 57 ± 3.21, *p* < 0.05), and Ig kappa constant (49 ± 2.08 vs. 43 ± 0.57, *p* < 0.01) were different between CD1 and C57BL/6 mice (Figure 4G – Immunoglobulins Graph).

## DISCUSSION

In this study, we showed that PRP intraovarian injection can improve blastocyst number and quality in POI mouse ovaries damaged by high doses of chemotherapy. Although our findings do not support an effect of PRP on follicle activation or growth, or the quantitative response to ovarian stimulation, we found that PRP injection increased the quality of oocytes, fertilization rates, and 2-cell embryos recovered in mice with POI.

This effect is likely due to the increase of local paracrine signaling through the released growth factors in the PRP-treated ovaries. PRP may promote the activation of various signaling cascades in the ovary, as it contains numerous growth factors that play crucial roles in cell proliferation, growth, and differentiation. These stimulatory effects could potentially lead to the activation of additional cascades indirectly, resulting from both direct and indirect mechanisms. Prior research lends support to the idea that PRP can directly induce paracrine signaling in the ovary through the action of plasma rich in growth factors [23, 24].

In women with the diagnosis of POI, extremely low ovarian reserve parameters are not always associated with the absence of follicles in the ovary. Clinical studies in POI patients showed that Hippo signaling disruption and Akt stimulation in POI ovaries can activate follicular growth; 5 of 27 women included in one study had successfully retrieved oocytes resulting in a livebirth after embryo transfer [25].

More recently, in a clinical study on PRP injection in patients with POI, 23 women conceived spontaneously (7.4%), and antral follicle development was observed in 70% of the rest of the study group (201/288). Of the 201 women who developed antral follicles, 82 generated embryos, 57 underwent embryo transfer and another 25 cryopreserved embryos to be transferred at a later stage. Therefore, autologous PRP injection seems to result in encouraging outcomes in patients diagnosed with POI [11]. In clinical studies, PRP seems to activate existing preantral and/or early antral follicles, and the number of remaining follicles in the ovaries of women with POI likely determines the extent of their response.

In patients with POR, the injection of PRP improved AFC, FSH, and AMH levels due to increased follicular recruitment and improved progression through follicular developmental stages [10]. Moreover, improvements in IVF parameters like the number of oocytes, fertilization rates and the number of blastocysts per cycle were observed, which could also be related to improvements in both oocyte quality and quantity [10]. Overall, women with POI and POR treated with an intraovarian injection of autologous PRP showed improved live birth rate and spontaneous conception [10, 11]. However, while these findings suggested that PRP treatment could be considered in patients with POI or POR, the effects of the PRP were not evaluated in an experimental model to delineate its mechanism of action.

Gonadotoxic treatments with alkylating agents have been widely used in animal studies. The high-standard drug dosage should reproduce the worst scenario of damage in the ovaries. The principal effects of cytotoxic agent toxicity in the ovarian stroma are related to cortical fibrosis, alteration in the vascularization and a reduction in the number of ovarian follicles [26]. Indeed, our study showed that PRP administration ameliorated the chemotherapy-induced damage of the ovarian stroma, contributing to generating an adequate niche for follicles to grow.

The most abundant population of follicles in the ovary are the dormant primordial follicles, which consist of an oocyte surrounded by a single layer of granulosa cells. Once activated, primordial follicles develop into primary follicles, secondary follicles, and ultimately may become antral follicles capable of producing a mature oocyte. Follicle activation might be induced through physiologic and non-physiologic pathways. Active substances such as growth factors and chemokines promote follicle activation and progression through stages of development. Some of these substances (transforming growth factor beta (TGF-β), insulin-like growth factor (IGF), platelet-derived growth factor (PDGF), epidermal growth factor (EGF), basic fibroblast growth factor (bFGF), vascular endothelial growth factor (VEGF)), cytokines (interleukin 1 beta (IL-1β), IL-8) are present in PRP and may help to explain the follicular activation and growth that occurs following intraovarian PRP injection [12, 27]. Indeed, the analysis of the mice PRP proteome demonstrated that platelets express numerous glycoprotein, integrin and G- protein-coupled receptors that bind to a myriad of soluble and matrix proteins and molecules, resulting in tightly orchestrated intracellular signaling related to angiogenesis, regeneration and immune pathways. Thus, the presence of all these growth factors with potential positive effects in both the stroma and follicles might underlie the clinical results reporting quantitative and qualitative improvements of folliculogenesis in both POR and POI patients. However, while our models do not reproduce the increased response to ovarian stimulation after intraovarian PRP injection, they suggest an increased quality of blastocysts.

Several authors also claimed qualitative effects of PRP on oocyte and embryo development, but these findings are still preliminary and limited to small case series and reports [9, 27–28]. Thus, we aimed to assess those outcomes related to quality in our models. Our results showed that in both the POR and POI animals, PRP administration increased oocyte quality, fertilization rate, and blastocyst formation rate leading to increased numbers of good-quality blastocysts after *in vitro* culture. The alkylating drugs used to generate our models reduced the ovarian reserve but also resulted in impaired oocyte quality, as damage to the DNA of oocytes cells, being double-strand breaks the most deleterious form of damage for germ cells [29]. Nevertheless, prophase-arrested oocytes, retain the ability to repair exogenous DNA damage by homologous recombination, especially those enclosed in primordial follicles [30]. As oocyte quality is not only related to the nucleus but also to cytoplasmic status [31], it may be possible that among all the regenerative growth factors contained in PRP, some of them exerted a positive effect allowing damaged oocytes to be repaired and develop to generate good quality embryos. The improved oxygen perfusion after intraovarian injection of platelet-derived growth factors could also promote improved intrafollicular oxygenation and better ooplasm quality. Subsequent recovery of mitochondrial function could be another pathway by which rescue of blastocyst quality is attained [32].

Altogether, our findings suggest a possible effect of the local injection of PRP on blastocyst formation and quality. These insights support the hypothesis of possible beneficial effects of PRP in a subpopulation of patients with diminished ovarian reserve.

## Supplementary Materials

Supplementary Figures

Supplementary Table 1
