# Supplementary material for: Intraovarian PRP injection improves oocyte quality and early embryo development in mouse models of chemotherapy-induced diminished ovarian reserve
Source: Aging (Albany NY). 2024 Sep 13;16(17):12123–37. doi: 10.18632/aging.206099 (PMC11424580; doi:10.18632/aging.206099)
Supplement: Supplementary Figures [file aging-16-206099-s001.pdf]

SUPPLEMENTARY FIGURES

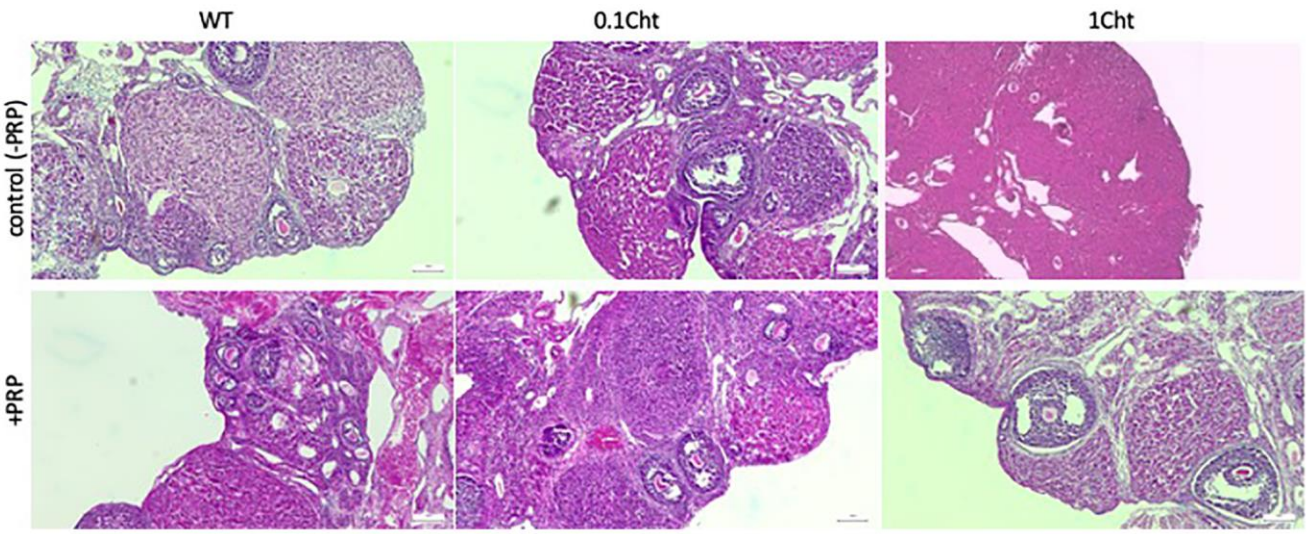

Supplementary Figure 1. Histological assessment of ovarian stroma. Hematoxilin-Eosin high magnification images of ovarian stroma and follicles from the wild type, 0.1ChT and 1ChT models.

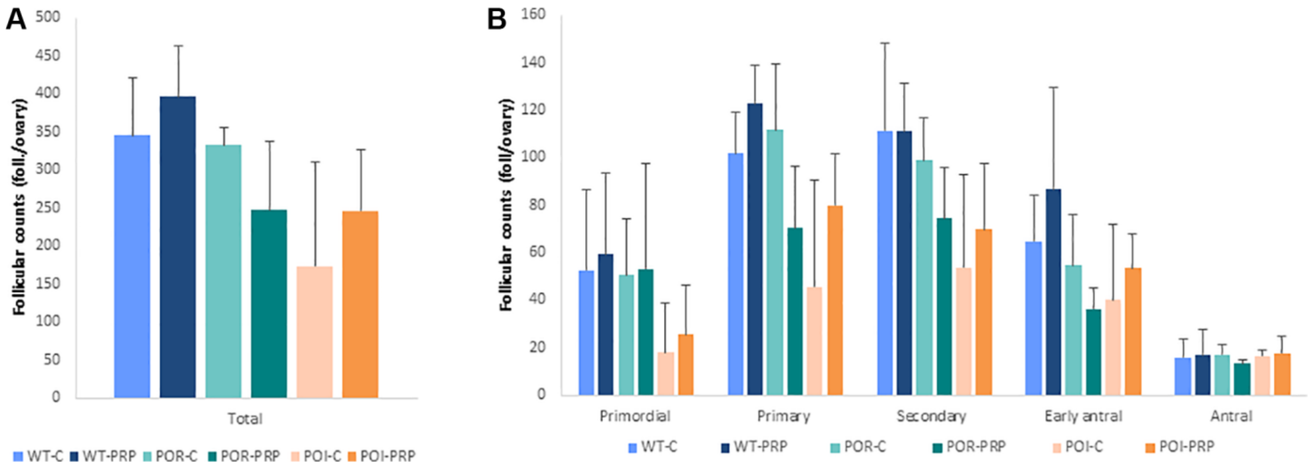

Supplementary Figure 2. Follicular counts in the CD1 model. (**A**) Total follicle counts. (**B**) Follicle subpopulations. Data are presented as mean ± SD. Follicles per ovary; $^{*}p < 0.05$.

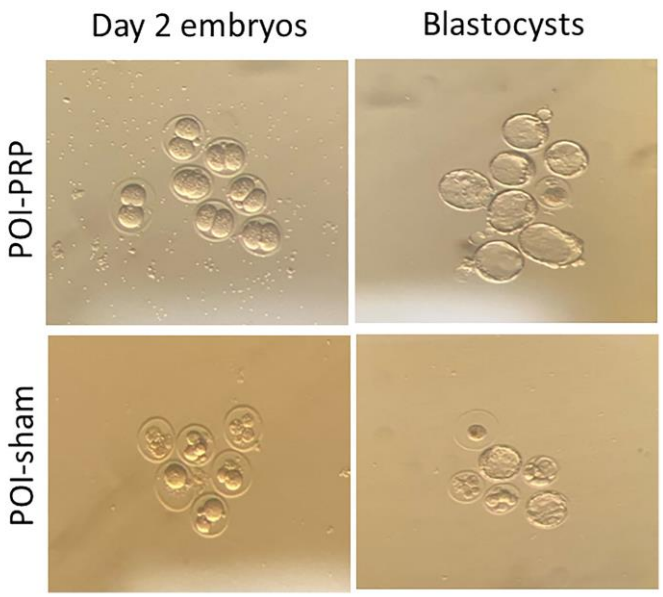

Supplementary Figure 3. Images of Day 2 embryos and blastocyst obtained from the POI-PRP and the POI-sham groups in the CD1 model.

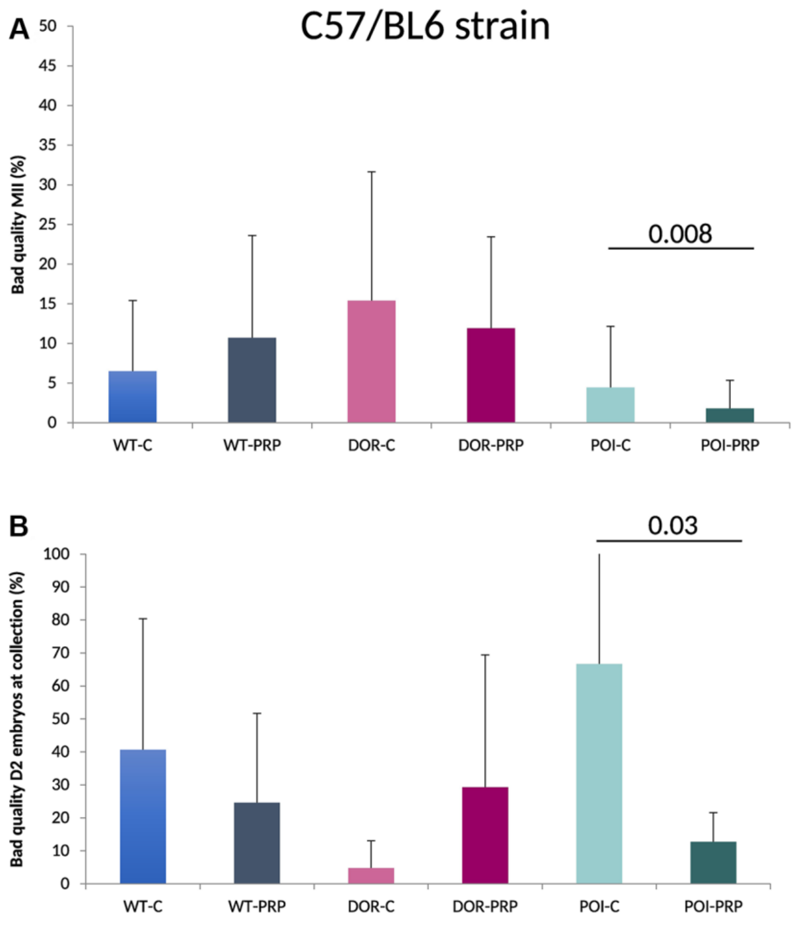

Supplementary Figure 4. Oocyte and embryo quality at collection in the C57/BL6 strain. (**A**) Percentage of bad quality MII oocytes at recovery. (**B**) Percentage of bad quality Day 2 embryos at collection.
