# Supplementary material for: Intraovarian PRP injection improves oocyte quality and early embryo development in mouse models of chemotherapy-induced diminished ovarian reserve
Source: Aging (Albany NY). 2024 Sep 13;16(17):12123–37. doi: 10.18632/aging.206099 (PMC11424580; doi:10.18632/aging.206099)
Supplement: Supplementary Table 1 [file aging-16-206099-s002.pdf]

SUPPLEMENTARY TABLE

Supplementary Table 1. Chemotherapy effects in the ovarian reserve of the C57/Bl6.

C57BL/6 foll./ovary	WT	POR	POI
**Primordial**	54.3 ± 18.9	44.3 ± 27.6	0.3 ± 0.6*
**Primary**	113.7 ± 38.4	93.7 ± 37.2	36.7 ± 2.5*
**Secondary**	108.0 ± 42.2	100.0 ± 53.0	25.3 ± 17.1*
**Early antral**	4.7 ± 3.5	7.3 ± 3.1	3.0 ± 2.6
**Antral**	11.0 ± 3.6	8.3 ± 2.5	8.3 ± 7.2
**Total**	291.7 ± 97.1	253.7 ± 122.7	73.7 ± 22.1*

*$p < 0.05$, using Kruskal-Wallis in the comparison as indicated and Mann-Whitney U-tests for two-by-two comparisons.
